# Functional Alterations of Postcentral Gyrus Modulated by Angry Facial Expressions during Intraoral Tactile Stimuli in Patients with Burning Mouth Syndrome: A Functional Magnetic Resonance Imaging Study

**DOI:** 10.3389/fpsyt.2017.00224

**Published:** 2017-11-06

**Authors:** Atsuo Yoshino, Yasumasa Okamoto, Mitsuru Doi, Go Okada, Masahiro Takamura, Naho Ichikawa, Shigeto Yamawaki

**Affiliations:** ^1^Department of Psychiatry and Neurosciences, Division of Frontier Graduate School of Biomedical Sciences, Hiroshima University, Hiroshima, Japan; ^2^Department of Dental Anesthesiology, Hiroshima University, Hiroshima, Japan

**Keywords:** chronic pain, burning mouth syndrome, postcentral gyrus, tactile, anger

## Abstract

Previous findings suggest that negative emotions could influence abnormal sensory perception in burning mouth syndrome (BMS). However, few studies have investigated the underlying neural mechanisms associated with BMS. We examined activation of brain regions in response to intraoral tactile stimuli when modulated by angry facial expressions. We performed functional magnetic resonance imaging on a group of 27 BMS patients and 21 age-matched healthy controls. Tactile stimuli were presented during different emotional contexts, which were induced *via* the continuous presentation of angry or neutral pictures of human faces. BMS patients exhibited higher tactile ratings and greater activation in the postcentral gyrus during the presentation of tactile stimuli involving angry faces relative to controls. Significant positive correlations between changes in brain activation elicited by angry facial images in the postcentral gyrus and changes in tactile rating scores by angry facial images were found for both groups. For BMS patients, there was a significant positive correlation between changes in tactile-related activation of the postcentral gyrus elicited by angry facial expressions and pain intensity in daily life. Findings suggest that neural responses in the postcentral gyrus are more strongly affected by angry facial expressions in BMS patients, which may reflect one possible mechanism underlying impaired somatosensory system function in this disorder.

## Introduction

Burning mouth syndrome (BMS) is associated with an intense, chronic intraoral burning sensation in the mouth with no mucosal lesions or other clinical signs and symptoms cannot be fully explained by medical test findings (1). It has been shown that the disorder involves abnormal sensory perception of tongue mucosa due to changed intraoral somatosensory system function, including a reduction in the epithelial density of small fiber endings (2). Although these findings suggest that BMS may be a peripheral neuropathic pain state, some studies have found no difference in sensory or pain thresholds between BMS patients and controls (3, 4). For instance, Kaplan et al.’s report suggested that thermal and pain thresholds of BMS patients during tongue stimulation did not differ from those of healthy controls (4), and the pathophysiology of BMS in the intraoral somatosensory system is not clearly understood.

A few functional magnetic resonance imaging (fMRI) studies have examined functional reorganization in BMS patients. For example, Albuquerque et al. reported that BMS patients exhibited distinct activation in the brain regions such as the precuneus and precentral gyrus during painful stimuli of the trigeminal nerve and showed a higher association between these activities and psychological distress (5). In their examination of brain responsivity to noxious heat stimulation on the palm or lip, Shinozaki et al. found that the cingulate cortex appeared to be involved in specific pain processing in BMS patients (6).

To date, research suggests that BMS has many psychological as well as physiological aspects, and convincing evidence for psychological involvement in the etiology of BMS has come from clinical studies (7–10). Prior reports have indicated that BMS patients were angrier, more anxious, and more depressed relative to controls (1, 10). In BMS patients, the intensity of somatic symptoms, including sensory perception in orofacial pain, has been associated with negative emotion (11–13). Overall, these findings support the view that BMS is associated with dysfunctional somatosensory mechanisms affected by psychological factors. However, prior studies of emotion modulation and sensory processing such as tactile perception in BMS patients have not demonstrated a causal relationship for neural mechanisms. Thus, we examined how negative emotion affects intraoral subjective somatosensory (tactile stimuli) and associated brain mechanisms in patients with BMS using fMRI. Our previous studies used sad facial expressions to induce emotional contexts (14, 15). In this study, angry facial expressions were adopted since anger has been identified as a more important emotional factor of deteriorated chronic pain severity than other negative emotions, especially sadness (11). It also seems that anger would be a key modulator of somatosensory perception in orofacial pain due to greater general physiological arousal (13).

Changes in postcentral gyrus activation have an important role in modulating tactile activity (16–21). Our previous magnetoencephalography study showed that sadness can enhance one’s subjective pain perception and increase postcentral gyrus activity during pain processing in healthy volunteers (15). Several laboratories have also demonstrated that sensory perception during unpleasant stimuli resulted in enhanced activity of the somatosensory system (22, 23).

Based on these findings, we hypothesized that somatosensory cortical activation changes in tactile stimuli modulated by angry facial expressions would be more strongly in patients with BMS than with controls.

## Materials and Methods

### Participants

The participants were 27 patients with BMS (21 women, mean age = 44.8 ± 12.0 years) and 21 gender- and age-matched control subjects (18 women, mean age = 46.3 ± 10.7 years). These age and gender characteristics were consistent with the previous epidemiology studies of BMS (1, 24). All participants were Japanese and right-handed. Patients were recruited from an outpatient dental anesthesia department at Hiroshima University Hospital. The diagnosis of BMS was made according to the classification of the International Headache Society by the same, trained dental anesthesiologist (author Mitsuru Doi) with more than 10 years of experience (25). Exclusion criteria for BMS included known causes of oral burning-like pain, such as no vitamin B12 deficiency, diabetes, anemia, thyroid disease, established neurological diseases (e.g., Parkinson’s disease), a past history of surgery or radiation to the head and neck region, or candidiasis. We also excluded participants with any current psychiatric comorbidity. Normal control participants were recruited from a non-clinical population. The control participants endorsed no chronic pain problems and had no history of psychiatric disorders. All participants gave their written informed consent before participation, according to protocol approved by the Ethics Committee of Hiroshima University.

### Clinical Assessment

#### Pain Characteristics

A visual analog scale (VAS) was used as a self-report measure of pain intensity in daily life.

#### Psychometric Evaluation

Participants completed the following questionnaires: the Beck Depression Inventory-Second Edition (BDI-II) (26), the State-Trait Anxiety Inventory (STAI) (27). The STAI includes two scales to differentiate anxiety related to a transitory or situational state (STAI-S) and trait anxiety (STAI-T).

### Experimental Paradigm and Stimuli

**Figure 1:**
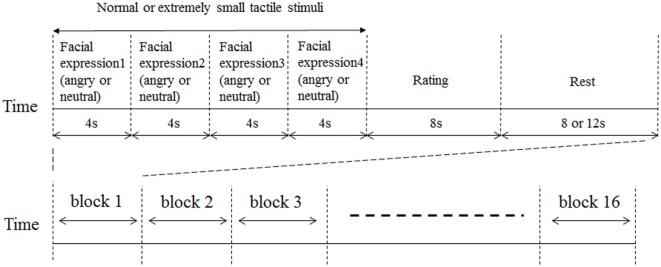
Schematic representation of experimental design. Facial expressions were presented for 4 s. The same emotion (angry or neutral) was represented four times sequentially in different randomly selected faces. For half of the randomly selected facial stimulus trials, tactile stimuli were delivered from presentation onset of the facial stimulus. For the other half of the facial stimuli, extremely small tactile stimuli were delivered. Tactile stimuli were delivered while the facial stimuli were presented. An 8 or 12 s rest period was inserted between each block of trials. Immediately after the presentation of tactile stimuli, participants were instructed to rate the average level of tactile intensity across the 8 s using a numeric rating scale (NRS) ranging from 0 to 10. Participants pushed a button to stop the bar moving between 0 and 10 to rate the intensity of their pain perception.

We conducted this experiment from January 2014 to August 2016. A schematic representation of the experimental block design is shown in Figure 1. Facial expressions were presented for 4 s. The same emotion was represented four times sequentially *via* different randomly selected faces. Such stimuli have been employed in many previous functional neuroimaging studies that examined neural responses to emotional stimuli (28–30). Tactile stimuli were delivered while facial stimuli were presented. This experimental design was based on our previous studies (14, 31). For half of the randomly selected facial stimulus trials, tactile stimuli were delivered beginning from the time of presentation onset of the facial stimulus. For the other half of the facial stimuli, extremely small corresponding tactile stimuli were delivered as participants were unable to feel. Each block was composed of four facial pictures with the same emotional valence (angry or neutral), tactile stimuli of the same intensity, a rating activity, and a rest period. Each block was 32 or 36 s in duration. The participants rated the average intensity of the tactile stimuli at the end of each block using a numeric rating scale (NRS) (0–10) projected onto the same screen for 8 s. For all participants, ratings scores of extremely small tactile stimuli in all conditions were 0. The entire paradigm comprised a sequence of 16 randomized blocks (4 blocks for each condition) and the total experimental duration was approximately 9 min. The fMRI testing sequence was performed at the same time of day (4:00 p.m. to 5:30 p.m.) for all participants to control for general temporal changes of symptoms in BMS (e.g., more severe complaints in the evening). The order of experimental conditions was counterbalanced across participants to mitigate order effects.

One pair of dish-type electrodes (diameter = 6 mm) was fixed to the right corner of the lip and oral mucosa across the oral cavity, and electrodes in each pair were placed 10 mm apart. Electrical stimuli consisted of 2 Hz constant-current biphasic pulses of 100 ms duration (NS-101 stimulator, Unique Medical, Tokyo, Japan). We established the stimulus current intensities for tactile stimuli (2.4 mA) and extremely small tactile stimuli (0.02 mA) and a preliminary experiment conducted before this study.

We used pictures of human faces as emotional stimuli, consistent with those used in previous functional neuroimaging studies (28–30). We used angry and neutral facial expressions to induce different emotional contexts while the participants were exposed to the tactile-inducing stimuli. Basically, it has been suggested that there is distinct neural network which is induced by each basic facial expression such as anger, fear, and sadness (32). Eight angry or eight neutral facial expressions displayed by eight different Japanese individuals (four females and four males) were taken from a standardized series of stimuli (33) and were presented for 4 s each per facial image. During fMRI recording, participants were instructed to imagine how the person depicted in each image felt when the image appeared on the screen. An MR-compatible back projection screen (Silent Vision SV-6011; Avotec, USA) was used to present the facial stimuli.

### Behavioral Data Analysis

Subjective tactile intensity ratings were analyzed using two-way repeated measures ANOVAs performed using SPSS version 16.0 with group (patients versus controls) as a between-subjects factor and emotional context (anger versus neutral) as within-subjects factors. Individual differences were controlled by using BDI-II, STAI-S, and STAI-T scores as covariates, in consideration of the modulatory effects of depression and anxiety on tactile sensitivity. Data were also examined using Bonferroni *post hoc* tests performed using SPSS version 16.0. Furthermore, correlations were examined between the anger-specific tactile rating scores and the clinical assessments including VAS, BDI-II, and STAI. The anger-specific tactile rating scores were defined by subtracting rating scores in the neutral condition from the angry condition. We analyzed the anger-specific rating scores by using two-sample *t* tests to determine between-groups differences.

### fMRI Acquisition

The fMRI procedure was performed using a Magnex Eclipse 3T Power Drive 250 (Siemens, Munich, Germany). A time course series of 366 scans was acquired using T2*-weighted, gradient echo, echo planar imaging sequences. Each volume consisted of 28 slices with a slice thickness of 4 mm with no gap and covered the entire cerebral and cerebellar cortices. The time interval between two successive acquisitions of the same image (TR) was 4,000 ms. Echo time (TE) was 46 ms and the flip angle was 90°. Field of view (FOV) was 256 mm and matrix size was 64 × 64, resulting in voxel dimensions of 4 mm × 4 mm × 4 mm. Scan acquisition was synchronized to the onset of each trial. After functional scanning, structural scans were acquired using a T1-weighted gradient echo pulse sequence (TR = 2,160 ms; TE = 3.93 ms; flip angle = 15°; FOV = 256 mm; voxel dimensions of 1 mm × 1 mm × 1 mm) to facilitate localization.

### fMRI Analysis

Image processing and statistical analyses were carried out using Statistical Parametric Mapping (SPM8) software (Wellcome Department of Cognitive Neurology, London, UK). The first three volumes of each fMRI acquisition were discarded because the MRI signal was unsteady. Each set of functional volumes was realigned to the first volume. A slice timing correction was performed on the model slice to correct for sequential sampling of the brain in the slice direction. Volumes were spatially normalized to a standard template based on the Montreal Neurological Institute reference brain, and smoothed using an 8-mm FWHM Gaussian kernel.

For the statistical analysis, subject-specific *t*-contrast images were calculated for the tactile effects using the general linear model (first-level analysis). For each participant, the preprocessed data were assigned to the following four conditions in the model specification: (1) tactile during angry facial images, (2) extremely small tactile during angry facial images, (3) tactile during neutral facial images, and (4) extremely small tactile during neutral facial images. Brain activations during tactile stimulation was defined by subtracting extremely small tactile during facial images conditions from tactile during facial images conditions, and these contrasts were entered into the second-level analysis. Group-level analyses were performed according to a random-effects model. A one-sample *t*-test was conducted to detect tactile-induced activity in all participants including the BMS and healthy control groups and two-sample *t* tests were conducted to detect between-group differences. Initially, regions of tactile activation common to all subjects were determined using a whole-brain one-sample *t*-test as a region of interest (ROI) prior to two-sample *t* test analysis. The degree of activation was calculated by averaging across all two emotional conditions. Second, a two-sample *t* test was carried out with ROI-based methodology using all of the voxels in each ROI. The BOLD signal changes involved in modulation by anger stimuli were defined in the angry > neutral contrast. Individual differences were controlled by using BDI-II, STAI-S, and STAI-T scores as covariates, in consideration of the modulatory effects of depression and anxiety on tactile sensitivity. Voxel-level thresholds were *p* (uncorrected) < 0.001, and cluster size thresholds were *p* (FWE corrected) < 0.05. Furthermore, we examined the correlations between the BOLD signal changes involved in modulation by anger stimuli and the behavioral data, such as the anger-specific tactile rating scores or clinical assessments including the BDI-II and STAI.

We also conducted SPM8’s simple regression analysis to examine correlations of individual difference scores such as anger-specific tactile rating scores and VAS with anger-specific BOLD signal changes for all of the voxels in each ROI as brain regions of tactile activation common to all participants. Voxel-level thresholds were *p* (uncorrected) < 0.001, and cluster size thresholds were *p* (FWE corrected) < 0.05.

## Results

### Participant Characteristics

Table 1 shows detailed demographic and clinical characteristics of the participants.

**Table 1:** Demographic and psychometric variables of patients and controls.

	Burning mouth syndrome (*n* = 27)	Controls (*n* = 21)	*T*_score_
**[Demographic variables]**			
Age	44.8 ± 12.0	46.3 ± 10.7	0.5^ns^
Female/male	21/6	18/3	0.5^ns^
Pain duration (months)	61.9 ± 43.0	–	–
Rating of pain in daily life (VAS)	4.6 ± 1.9/10	–	–
**[Psychometric variables]**			
BDI-II	11.8 ± 6.9	4.8 ± 5.7	3.8*
**STAI**			
STAI-T	48.6 ± 11.7	38.3 ± 8.6	3.3*
Trait	49.0 ± 13.3	39.8 ± 11.1	2.6*
PCS	26.9 ± 8.2	16.9 ± 12.9	3.3*

ns, not significant, *p_two-sided_ < 0.05 (two-sample *t*-test).

*BDI-II, Beck Depression Inventory-Second Edition; VAS, Visual Analog Scale; STAI, State-Trait Anxiety Inventory; PCS, Pain Catastrophizing Scale*.

### Behavioral Results

**Figure 2:**
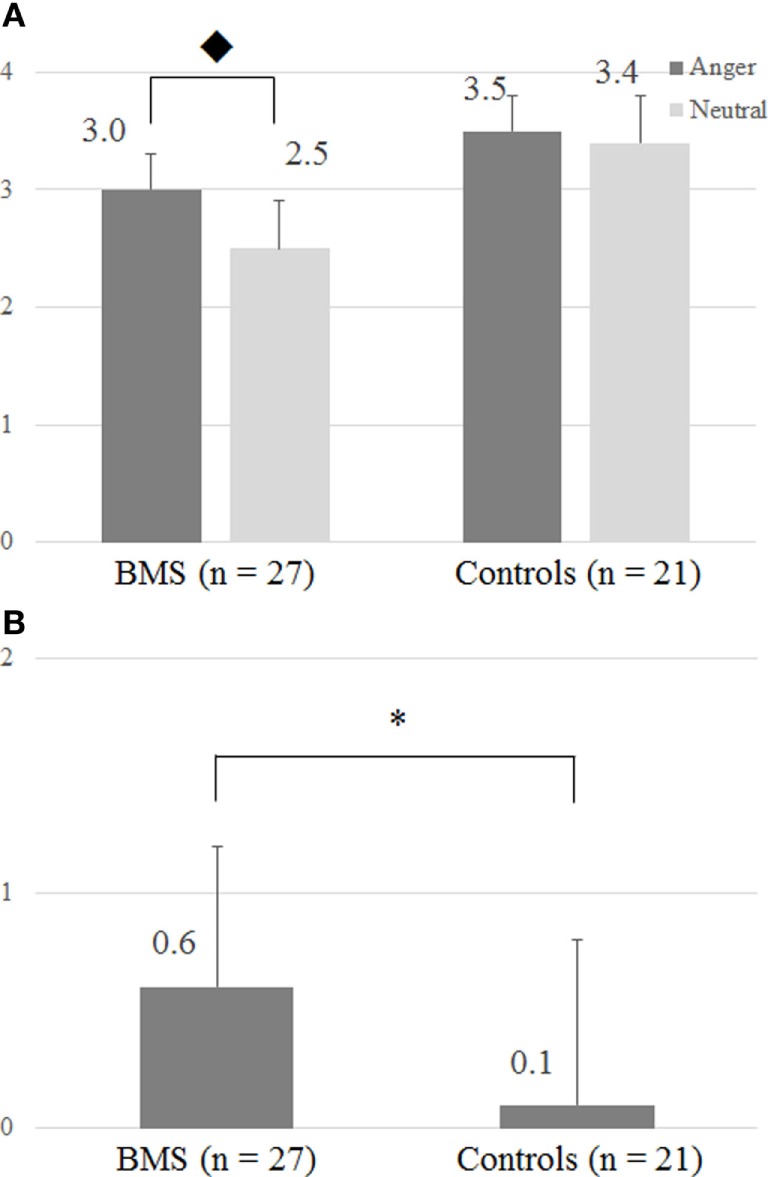
**(A)** Intensity of tactile perception by the differences of facial images. **(B)** Anger-specific tactile rating scores. The *y*-axis in panels **(A,B)**, respectively, represents a numeric rating scale (NRS) rating score and an anger-specific tactile rating score which was defined by subtracting NRS rating scores in the neutral condition from the angry condition. ◆ The two-way ANOVA (Group × Emotion) revealed significant interactions (*F*1, 46 = 4.56; *p* < 0.05). In patients, ratings in the tactile anger condition differed significantly from those in the neutral condition (Bonferroni *post hoc t* test, *p* = 0.05). Anger-specific tactile rating scores were defined by subtracting rating scores in the neutral condition from the angry condition. *p < 0.05 (two-sample *t* test).

Participants reported different tactile intensity ratings across the emotional context conditions. The two-way ANOVA (Group × Emotion) revealed a significant interaction (*F*1, 46 = 4.56, *p* < 0.05) (Figure 2A), and for the patient group, tactile ratings in the anger condition were significantly higher than in the neutral condition (Bonferroni *post hoc t* test, *p* < 0.05). Moreover, the two-sample *t* test showed a significant difference between BMS patients and healthy controls (*p* < 0.05) in the anger-specific tactile rating scores (Figure 2B). There were no significant correlations between the anger-specific tactile rating scores and psychometric variables.

### fMRI Data

#### Brain Activation Involved in Tactile Perception for the Sample (One-Sample *t*-Test of “Tactile”)

**Figure 3:**
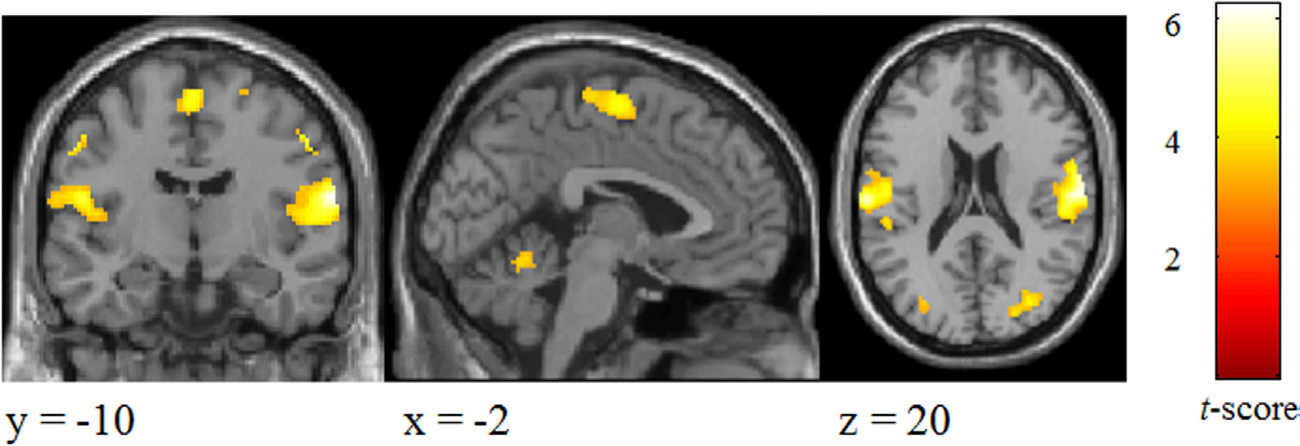
Brain areas activated by tactile stimuli for all two emotional conditions in participants.

Significant changes were detected mainly in the postcentral gyrus and superior parietal lobule (Figure 3; Table 2).

**Table 2:** Brain areas activated by tactile stimuli for all two emotional conditions in participants.

Brain regions	L/R	*x*/*y*/*z*	*t* Value	Cluster extent
Postcentral gyrus	R	64/−12/18	6.22	1,365
Postcentral gyrus	L	−64/−18/18	5.80	747
Postcentral gyrus	L	−56/−18/42	5.99	382
Supplementary motor area	L/R	0/−10/64	4.41	246
Superior parietal lobule	R	24/−58/56	4.90	945
Superior parietal lobule	L	−20/−66/52	5.35	868

*Uncorrected p < 0.001 and FWE-corrected cluster level p < 0.05*.

#### Differences in Tactile Processing Modulated by Anger Stimuli (Anger > Neutral Contrast) in the Brain Areas between Groups

There were no significant changes in activation during tactile stimulation under the all emotional conditions between patients and controls.

**Figure 4:**
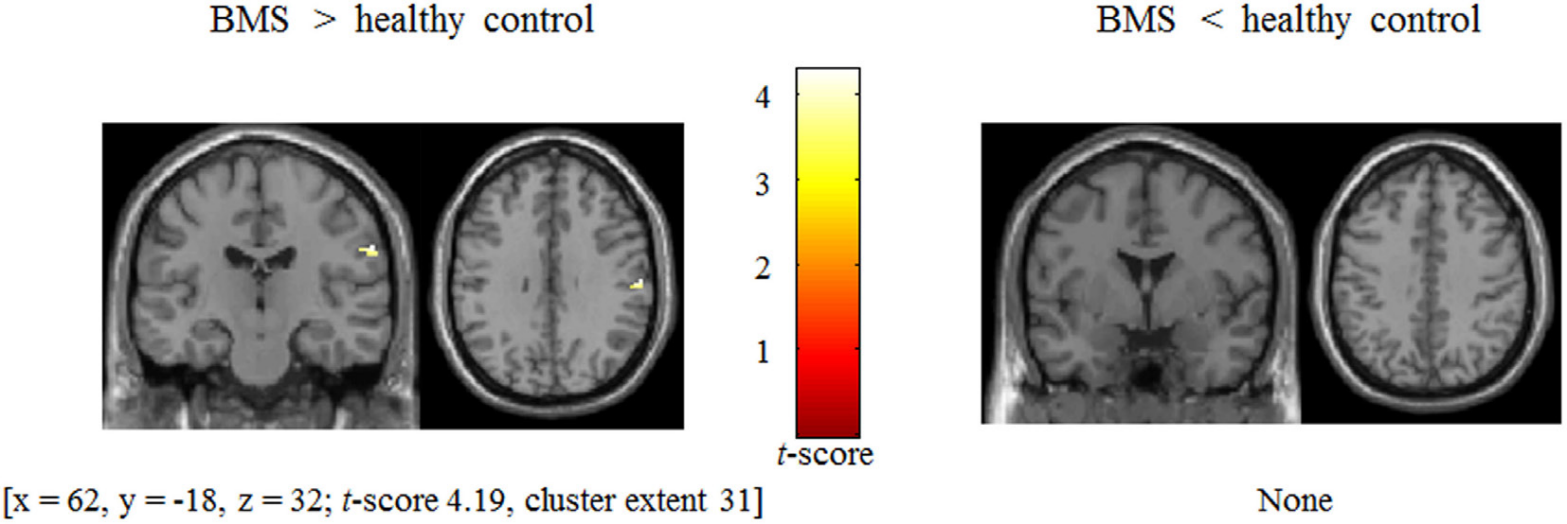
Difference in BOLD signal change between patients with burning mouth syndrome (BMS) and controls in postcentral gyrus during presentation of tactile stimuli modulated by angry emotion (anger − neutral).

BOLD signal changes (anger > neutral contrast) involved in modulation by anger stimuli in patients was associated with stronger postcentral gyrus activation (*x* = 62, *y* = −18, *z* = 32; *t*-score 4.19, cluster extent 31) [two-sample *t*-test, *p* (uncorrected) < 0.001 and cluster size thresholds were *p* (FWE corrected) < 0.05, Figure 4] in patients relative to controls.

There was a significant positive correlation between anger-specific BOLD signal changes and VAS in BMS patients in the postcentral gyrus which activated during anger > neutral contrast (*r* = 0.38, *p* < 0.05). There was a significant positive correlation between the anger-specific BOLD signal changes and anger-specific tactile rating scores in all participants (*r* = 0.29, *p* < 0.05). There were no significant correlations between the anger-specific BOLD signal changes and other psychometric variables. We did not also find any statistically significant correlations of individual difference scores such as anger-specific tactile rating scores and VAS with anger-specific BOLD signal changes for all of the voxels in each ROI as brain regions of tactile activation common to all participants.

## Discussion

Findings showed that, relative to controls, BMS patients exhibited significantly higher levels of subjective tactile ratings in the angry facial images relative to the neutral facial images as well as stronger activation changes during tactile stimuli modulated by angry facial images in the right postcentral gyrus. In both groups, changes in tactile rating scores by angry facial images were positively associated with changes in postcentral gyrus activation during tactile stimuli induced by the angry facial images. In the patient group, VAS scores were also positively linked with changes in postcentral gyrus activation during tactile stimuli induced by the angry facial images. Overall, this is the first fMRI study to examine the association between anger and dysfunctional somatosensory mechanism in BMS patients.

### Subjective Tactile Intensities

Subjective tactile intensities in the angry condition were significantly greater than those in the neutral condition for BMS patients relative to the control group. In chronic pain studies (11), we were able to replicate the between-group differences in perception rating associated with anger, and this study was the first to confirm that subjective tactile intensities were modulated by emotion in intraoral BMS patients.

### Differences in Brain Activation between Patients and Controls

At first, we have confirmed the activation of bilateral postcentral gyrus in both participants during the presentation of intraoral tactile stimuli. According to the “homunculus” model (34), intraoral sensation is mapped in the lower part of the postcentral gyrus, and our results were also similar to those of Jasper and Penfield. The results showed that there were no significant changes in activation during tactile stimulation under the all emotional conditions between groups. Previous experimental studies in somatic perception found no difference in sensory or pain thresholds between groups (3, 4), and these results may be demonstrated from the perspective of neuroimaging.

The present results were consistent with our hypothesis that changes in postcentral gyrus activation in tactile stimuli associated with anger would significantly increase in patients with BMS. Several pain-induced studies have demonstrated increased neural responses in pain–matrix network in patients with BMS relative to controls (5, 6). Prior research in our laboratory has also shown increased neural responses in chronic pain patients during pain perception when modulated by sadness (31). However, no studies of BMS patients to date have examined neural responses and subjective tactile perception when modulated by angry facial expressions during the presentation of intraoral stimuli. This study suggests that the angry emotional condition is associated with specifically enhanced tactile-related brain activation and somatic sensation in BMS, and we speculate that psychological factors are involved in the etiology of BMS. Many previous tactile-related studies have demonstrated that activation in the postcentral gyrus was changed by negative emotion (15–21), and we consider that the postcentral gyrus is one of the most important brain regions for pathophysiology of psychological factors in BMS. In this study, there were no differences in emotional brain processing areas such as the insula and the anterior cingulate cortex between groups. Further studies are needed to examine whether these regions are related to tactile modulation, including changes of task designs and of emotion.

Our results also revealed that the pain-related VAS scores in daily life were positively correlated with changes in postcentral gyrus activation during tactile stimuli in the angry condition for BMS patients. This finding suggests that clinical characteristics in BMS patients may be linked to the hypersensitivity of intraoral sensory perception associated with the angry emotional condition.

This study has several limitations. First, exclusion criteria for participants did not include all possible treatment effects that might influence perceptions of patients, such as the use of antidepressants. It has been reported that antidepressants produce changes in pain-related brain activity (35). However, it is not clear whether antidepressants influence effects in acute physical stimuli (36). Second, we could not rule out all treatment effects on brain activation that was observed in this study.

In conclusion, we found the distinctive activation of the postcentral gyrus in BMS patients while receiving tactile stimulation modulated by angry facial images. Relative to controls, patients showed more changes in activation induced by anger-context tactile stimuli in the right postcentral gyrus. This study has also revealed that, across both groups, there was a significant positive correlation between behavioral data (i.e., subjective tactile rating scores changed by angry stimuli) and postcentral gyrus activation modulated by angry facial images during the presentation of tactile stimuli. Scores on the VAS in daily life were positively associated with anger-changed postcentral gyrus activation during tactile stimuli in BMS patients. These results suggest that the modulatory function of somatosensory perception with regard to emotion may be impaired in BMS patients. In summary, the interaction between brain activity and emotional context associated with tactile stimuli may potentially play an important role in the pathophysiology of BMS.

## Ethics Statement

All participants gave their written informed consent before participation, according to protocol approved by the Ethics Committee of Hiroshima University. All procedures followed were in accordance with the ethical standards of the responsible committees on human experimentation (institutional and national) and with the Helsinki Declaration of 2013, and the appropriate revisions at the time of the investigation. Informed consent was obtained from all patients included in the study.

## Author Contributions

AY was involved in the experimental design, data collection, analysis of MRI data, and writing of the manuscript. MT, GO, and NI contributed to the analysis of MRI data. MD contributed to the data collection. YO and SY contributed in the experimental design and revision of the manuscript.

## Conflict of Interest Statement

AY has received prior research support from Eli Lily. There are no other disclosures to report.
